# Using Biological Feedback to Promote Health Behavior Change in Adults: Protocol for a Scoping Review

**DOI:** 10.2196/32579

**Published:** 2022-01-18

**Authors:** Kelli M Richardson, Ahlam A Saleh, Michelle R Jospe, Yue Liao, Susan M Schembre

**Affiliations:** 1 Department of Nutritional Sciences College of Agriculture and Life Sciences University of Arizona Tucson, AZ United States; 2 Arizona Health Sciences Library Tucson, AZ United States; 3 Department of Family and Community Medicine College of Medicine University of Arizona Tucson, AZ United States; 4 Department of Kinesiology College of Nursing and Health Innovation University of Texas at Arlington Arlington, TX United States

**Keywords:** monitoring, physiologic, biomarkers, feedback, psychological, health behavior, health promotion, biofeedback, health databases, health interventions

## Abstract

**Background:**

Many health conditions can be prevented, managed, or improved through behavioral interventions. As a component of health behavior change interventions, biological feedback is of particular interest given recent advances in wearable biosensing technology, digital health apps, and personalized health and wellness. Nevertheless, there is a paucity of literature to guide the design and implementation of interventions that incorporate biological feedback to motivate health behavior change.

**Objective:**

The goal of this scoping review is to deeply explore the use of biological feedback as a component of health behavior change interventions that target adults. The objectives of the review include (1) mapping the domains of research that incorporate biological feedback and (2) describing the operational characteristics of using biological feedback in the context of health behavior change.

**Methods:**

A comprehensive list of search terms was developed to capture studies from a wide range of domains. The studies to be included are randomized controlled trials published as primary research articles, theses, or dissertations targeting adults 18 years and older, who use biological feedback to change a health-related behavior. The following electronic databases were searched: Ovid MEDLINE, Embase, Cochrane Central Register of Controlled Trials, EBSCOhost, PsycINFO, and ProQuest Dissertations & Theses Global. The screening and data extraction process will be guided by the Joanna Briggs Institute Manual for Evidence Synthesis and conducted by trained reviewers.

**Results:**

Database searches were completed in June 2021. A total of 50,459 unique records were returned after the removal of 48,634 duplicate records. The scoping review is planned for completion in 2022.

**Conclusions:**

To our knowledge, this will be the first scoping review to map the literature that uses biological feedback as a component of health behavior change interventions targeting adults. The findings will be used to develop a framework to guide the design and implementation of future health behavior change interventions that incorporate biological feedback.

**Trial Registration:**

OSF Registries OSF.IO/YP5WA; https://osf.io/yp5wa

**International Registered Report Identifier (IRRID):**

DERR1-10.2196/32579

## Introduction

Historically, infectious diseases were the leading causes of death worldwide [1]. Medical advances were made to target infectious agents and successfully eradicate disease. Aside from COVID-19, health conditions including cardiovascular disease, cancer, and respiratory disease, all of which are affected by modifiable personal health behaviors, are the leading causes of death in developed countries [1,2]. Substance use, physical inactivity, and poor diet are examples of modifiable health behaviors that are causally associated with poor health outcomes [3,4]. Many health conditions can be prevented, managed, or even treated through interventions targeting these and other health-related behaviors [5,6]. However, the development of public health interventions aimed to improve health outcomes is complex, particularly in the context of advancing technology and science that serve to complement standard medical care [7,8]. For decades, health behavior change research has relied on behavioral theories, most commonly the transtheoretical model of change, theory of planned behavior, and social cognitive theory, to guide the development of effective health promotion interventions [9,10]. Although transformational, work in this field has mostly led to the development of comprehensive one-size-fits-all interventions to which not everyone responds favorably [11]. More recently, innovations in wearable biosensing technology as well as mobile and digital health have laid the foundation for moving from one-size-fits-all interventions toward personalized approaches to health and wellness [12-14]. Personalized interventions are tailored to an individual’s traits (eg, via genotyping) or state (eg, via metabotyping) with the goal of improving personal health–related outcomes. Despite the promise of such interventions, their design and implementation are complex, and they are yet to be optimized [15]. Research examining best practices for using and sharing biological data to optimize personal health–related outcomes, particularly in the context of motivating health behavior change (ie, biological feedback), represents a fundamental step toward developing effective personalized health and wellness interventions for health promotion.

Using a person’s biological data to choose an intervention that could have the greatest likelihood of success is not new. In fact, it is a relatively common practice in some fields of medicine, including genetic counseling, medical decision-making, and cancer treatment. However, we herein operationally define biological feedback as providing individuals with their biological data through direct communication (via an unblinded body-worn assessment device such as a heart rate monitor or a continuous glucose monitor) or indirect communication (via health coaches, patient educators, or messaging systems) to motivate health behavior change explicitly or implicitly for improving health-related outcomes. One type of biological feedback used in health behavior change interventions is biofeedback. Michie and colleagues define “biofeedback” as a behavior change technique (BCT) that “informs the person about their physiological or biochemical state using an external monitoring device to improve the adoption of health behaviors” [16]. An example of biofeedback as a BCT is the use of a heart rate monitor to achieve the prescribed exercise intensity as part of a physical activity intervention. It is important to note that biological feedback, as defined herein, and biofeedback as a BCT vary conceptually from the traditional mind-body therapy referred to as biofeedback. As a mind-body therapy, biofeedback is a technique that involves the use of electrical sensors to provide information about the body (eg, muscle contractions) to help people learn how to control bodily functions (eg, urinary incontinence) [17]. This form of biofeedback is most often used to treat or manage a range of clinical conditions often involving the autonomic nervous system, and it is not the focus here [18]. Instead, the goal of the planned scoping review is to deeply explore the use of biological feedback as a technique to motivate health behavior change. The findings will be used to guide the development of future health behavior change interventions that incorporate biological feedback.

The potential value of this work is exemplified by the highly novel but limitedly effective Food4Me trial. Food4Me was a 6-month randomized controlled trial (RCT) conducted across 7 European countries that emulated a real-life web-based personalized nutrition service where participants received 1 of 4 levels of personalized dietary advice (generalized, L0; based on dietary intake, L1; based on dietary intake + phenotype, L2; and based on dietary intake + phenotype + gene, L3) [19]. Additionally, those in the personalized feedback arms of the Food4Me trial were further randomized to receive low-intensity nutritional feedback (delivered at baseline, month 3, and month 6) or high-intensity feedback (delivered at baseline and months 1, 2, 3, and 6). The primary aim of the Food4Me trial was to determine if personalization of dietary advice helped people improve their diet quality (healthy eating index scores) in comparison with nonpersonalized conventional healthy eating guidelines [19]. A secondary aim was to compare high-intensity and low-intensity feedback to determine if they resulted in improved outcomes. Results showed no evidence that the addition of biological feedback on phenotypic and phenotypic plus genotypic information enhanced the effectiveness of the personalized nutrition advice [20]. Findings specific to feedback intensity showed that improvements in diet quality were greater in the high-intensity vs low-intensity feedback group at 3 months or when nutritional feedback was provided monthly (vs quarterly) [21]. Despite these findings, the Food4Me trial was among the first to show the positive outcomes of personalized dietary advice compared to conventional dietary advice. Since the completion of the Food4Me trial, there has been a substantial increase in related research initiatives worldwide, including the National Institutes for Health’s precision medicine and precision nutrition initiatives in the Unites States. Given the substantial financial investment into precision health that is being made in the United States and elsewhere, it is imperative that research aimed at optimizing the health-related outcomes of precision health interventions be conducted.

As a first step to harnessing the potential of biological feedback as a health behavior change intervention, we are conducting a scoping review to explore the historical and current use of biological feedback in health behavior change interventions that target adults. This type of review is necessary because to our knowledge, the only known review on this topic was published in 2002 [22]. It was an empirical review of 8 published RCTs that used biomarkers to educate individuals about their health status and disease risk to promote health behavior change. Findings were generally supportive and suggested that biological information related to harm exposure, disease risk, or impaired physical functioning increases the motivation to change behavior, particularly when there is evidence of physical damage or significant personal risk related to the behavior. However, significant effects on behavior change depended on the intensity of the concomitant treatment, similar to the Food4Me trial, and were only observed when a single biomarker was assessed on multiple occasions or when multiple biomarkers were assessed on a single occasion. Although the previous review provided evidence regarding the efficacy of using biological feedback to motivate health behavior change, a more comprehensive review is needed to learn how variable the use of biological feedback is as a first step toward determining the best method to implement future interventions. As such, the objectives of this scoping review are to (1) map the domains of research that incorporate biological feedback as a health behavior change intervention and (2) describe the operational characteristics for implementing biological feedback as a health behavior change intervention. Findings will be used to develop a framework to guide future health behavior change interventions that incorporate biological feedback. Further work will be done to examine the efficacy of using biological feedback to motivate health behavior change and improve health-related outcomes. The following questions will be answered as part of the scoping review:

1. Which public health domains are using biological feedback as a component of health behavior change interventions targeting adults (eg, diabetes, substance abuse)?
2. What are the targeted health behaviors (eg, diet, exercise, smoking) and outcomes (eg, weight loss, glucose stability) applicable to using biological feedback as a component of health behavior change interventions?
3. Which biological measures are being used for providing feedback (eg, body weight, carbon monoxide levels), and how are biological measurements obtained (eg, self-measurement, clinical)?
4. How is the feedback communicated (ie, on which platform and in which format)?
5. Which behavior change theories are cited, if any, as the foundation for using biological feedback to promote health behavior change?

## Methods

The proposed scoping review (OSF Registries OSF.IO/YP5WA; http://doi.org/10.17605/OSF.IO/YP5WA) will be guided by the Joanna Briggs Institute Manual for Evidence Synthesis [23]*.* The review process is being managed using DistillerSR (Evidence Partners), a software package used for systematic reviews and literature reviews.

### Types of Participants

Eligible studies will be those that target adults (18 years or older). Studies will be included regardless of the disease conditions of the participants. Studies targeting health behavior change only in infants, children, and adolescents will be excluded.

### Concept

This scoping review will consider RCTs that include biological feedback as a component of health behavior change interventions. RCTs meeting the following criteria will be selected: (1) Biological data reflecting a study participant’s physiological state or traits are collected. (2) The study participants are provided with their biological data through direct or indirect feedback. (3) The intent of providing feedback is to motivate health behavior change explicitly or implicitly. The core concept of the scoping review is to describe the historical and current landscape and methodology for using biological feedback in health behavior change interventions. We aim to include any measurable biological states and traits for which feedback can be provided.

### Context

All included studies must aim to change a health behavior. Here, health behavior is defined as “...behavior patterns, actions, and habits that relate to health maintenance, to health restoration, and to health improvement” [24]. The proposed scoping review will include all behaviors that are modifiable and can improve (or decline) health. Examples of health behaviors include diet, exercise, smoking cessation, medication adherence, and use of medical services [25]. In the context of the proposed scoping review, health behavior change must be the intended purpose for providing biological feedback (vs diagnostics). Studies using traditional forms of biofeedback as a mind-body therapy will be excluded, as this therapeutic technique most typically aims to directly modulate the disease or health condition as opposed to motivating health behavior change, though there may be some exceptions. Studies will be included regardless of the setting (ie, acute care, primary care, community).

### Types of Evidence Sources

Evidence sources will include published primary research articles, theses, and dissertations in any language. There will be no limits set on the year of publication unless deemed evident by a sudden increase in eligible literature by year. If no trend is observed, the time frame will remain open.

The search will be limited to RCTs. Though cohort studies, case-control studies, cross-sectional studies, case reports, conference abstracts, and papers could incorporate biological feedback, these studies will be excluded for reasons of feasibility. Evidence syntheses including scoping reviews, systematic reviews, and meta-analyses will also be excluded. Websites, blogs, and published letters will be excluded as well as incomplete works such as clinical trial protocols and other gray literature such as government reports and policy or issue papers. Retracted articles will also be excluded.

### Search Strategy

With the aid of a research librarian, terminology was identified to reflect 3 key components of the review, namely the biological measure, feedback modality, and intervention context. A search strategy was devised using controlled vocabulary and text words in MEDLINE and then adapted to the other databases. The electronic databases that were searched included Ovid MEDLINE and Epub Ahead of Print, In-Process, In-Data-Review & Other Non-Indexed Citations, Daily and Versions, Embase, Cochrane Central Register of Controlled Trials, EBSCOhost, PsycINFO, and ProQuest Dissertations & Theses Global. The Ovid MEDLINE database search strategy is provided in Multimedia Appendix 1. After drafting an initial Ovid MEDLINE search strategy, the identified records from the databases were searched to examine if self-identified records that were known to meet the eligibility criteria were captured using this search strategy. If they were not, we modified the search to include the newly identified terms from known eligible records. Additional search methods included examining the reference lists of relevant scoping and systematic reviews and meta-analyses to find any additional eligible primary research articles.

### Source of Evidence Selection

Prior to initiating the screening process, reviewers were trained via pilot tests using the screening form(s). Modifications to the screening questions were made during this time to ensure clarity for all reviewers.

The review of records is being conducted using 2 levels of screening. In the first level of screening, the trained reviewers independently screen the titles and abstracts for initial eligibility. Records that do not describe primary findings from RCTs targeting human adults will be excluded at this level. Quality control measures that include an additional reviewer and the DistillerSR artificial intelligence feature are being used to review excluded records for erroneous exclusion. Records confirmed to have been erroneously excluded will be included and subjected to the next level of review. In the second level of screening, trained reviewers will (1) confirm inclusion based on the first level of screening and (2) review abstracts for information regarding the use of biological feedback to motivate health behavior change. The decision to conduct this level of review via abstract screening only was informed by a pilot test where the accuracy of excluding articles via abstract screening vs full text screening was examined. A total of 34 of 100 records that passed the first level of screening were reviewed by both methods. Results indicated that 22 of the 23 articles (96%) were accurately excluded by abstract screening only. Therefore, we deemed this method acceptable. An exception to this approach was made for studies that implemented “self-monitoring” or “self-management” strategies or when feedback on health risk was provided to the study participants. For these articles, the full text was screened to determine whether a biological measure was used. Second-level screening will be conducted using double data entry. Data entry conflicts will be reviewed and resolved by an independent reviewer. Records passing second-level screening will be subjected to data extraction.

### Data Extraction

Data extraction forms will be designed to collect data relevant to the aims of the scoping review. Key information to be extracted will aim to describe the implementation of biological feedback as a component of a health behavior change intervention targeting adults. This will include, but may not be limited to, the following:

1. Author(s)
2. Title
3. Year
4. Biological measure (eg, blood pressure, carbon monoxide, genetics)
5. Targeted behavior (eg, alcohol use cessation, diet, physical activity)
6. Targeted health-related outcome or intent of intervention (eg, glycemic control, weight management, mental health improvement)
7. Domain (eg, cancer, diabetes, substance abuse)
8. Method of obtaining biological measures (eg, self-measurement, clinic)
9. Feedback platform (eg, in person, monitoring device, telephone call)
10. Format of feedback (eg, number, graph, image)
11. Behavior change theory (eg, health belief model, theory of planned behavior)

A draft of the data extraction form is presented in Multimedia Appendix 2. If useful data that we did not plan to extract are available in the records, the data extraction form will be revised, and these additional data will be extracted from previously reviewed records. Additionally, ineligible articles may be identified during the data extraction process for reasons described in the first and second levels of screening. In such cases, the reason for exclusion will be noted and data from those records will not be included in the analysis of the evidence.

### Analysis of the Evidence

As the aim of this scoping review is to map the domains of research that incorporate biological feedback, study results presented in the records will not be analyzed. Instead, summary data related to the aim will be synthesized descriptively.

### Presentation of the Results

The PRISMA (Preferred Reporting Items for Systematic Review and Meta-Analysis) 2020 flow diagram will be used to present the selection process [26]. This includes the number of records identified, number of records after duplicates are removed, number of records after eligibility screening, and final included number of records. Findings of the included records will be presented through evidence mapping and descriptive summaries.

## Results

The database search was completed in June 2021. The search yielded 99,093 records. All results were originally exported into EndNote X9 (Clarivate Analytics) and deduplicated by the research librarian. There were 48,510 duplicate records identified in EndNote. The resulting 50,583 records were imported into DistillerSR for review. An additional 124 duplicate records were identified in DistillerSR and removed. As a result, the search produced 50,459 unique records. The scoping review is planned for completion in 2022.

## Discussion

A sizable but uncharacterized body of literature has shown the potential for the use of biological feedback as a component of health behavior change interventions. The proposed scoping review aims to explore the breadth of domains using biological feedback as a health BCT in interventions targeting adults. More than 200 search terms characterizing a wide variety of biological measures and modes of delivering feedback in various health-promoting contexts were derived to fulfill this goal. To our knowledge, this will be the first scoping review to map the literature in this area with the intent of informing future health behavior change interventions that incorporate biological feedback.

The proposed review has some limitations. One of them is that there is no consistent terminology for the use of biological feedback in health behavior change interventions. For instance, “blood glucose self-monitoring” is a type of biological feedback but the term “biological feedback” is not explicitly stated in the bibliographic and abstracting information. Due to inconsistencies in terminology, a compilation of over 200 terms reflecting the collection of biological data, the provision of feedback either directly via body-worn sensors or indirectly by an external agent or software, and the contexts in which behavioral interventions can be delivered were used in the search strategy to capture a majority of the biological feedback studies. It is possible that the resulting list of search terms did not return all relevant records. In cases where additional search terms are identified through reviewing the returned records or relevant scoping or systematic reviews, these terms will be added to the search strategy to identify additional records. Another limitation is that for feasibility reasons, this scoping review will include only RCTs. However, with the breadth of our search strategy, we will still capture a considerable number of studies spanning many domains of research. Therefore, this limitation should not negatively impact our ability to describe the use of biological feedback as a component of behavior change interventions. Moreover, we will not be including intervention studies targeting infants, children, or adolescents. As such, our findings will be generalizable to only adult populations. Lastly, due to feasibility issues, in our primary screening, only the titles and abstracts of the returned records will be reviewed (vs full text screening). Consequently, studies may be erroneously excluded. However, the decision to screen only the titles and abstracts was informed by a pilot test that confirmed an accuracy level exceeding 95% for this approach. Therefore, it is unlikely that this approach will negatively affect the objectives of our review. Despite these limitations, this scoping review represents a fundamental first step toward developing effective precision health interventions.

The methods outlined above were developed specifically to capture a wide range of health-promoting interventions that incorporate the use of biological feedback to motivate behavior change. The results will summarize the characteristics of this research, including the domains, targeted health behaviors and health-related outcomes, biological measures and forms of measurement, platforms and content on which feedback was provided, and behavior change theories used in interventions incorporating biological feedback. Future research will use the findings from this scoping review to generate ideas for primary research aimed to optimize the implementation of biological feedback to produce meaningful health behavior changes in public health interventions. Additionally, results from this scoping review and subsequently planned systematic reviews will be used to develop a framework to guide the use of biological feedback in future health behavior change interventions.

## Appendix

Multimedia Appendix 1 Search strategy.

Multimedia Appendix 2 Preliminary data extraction form.

## Abbreviations

BCT: behavior change technique
RCT: randomized controlled trial

## References

[1] Centers for Disease Control Prevention (CDC) (1999). Control of infectious diseases. MMWR Morb Mortal Wkly Rep.

[2] Ahmad FB, Anderson RN (2021). The leading causes of death in the US for 2020. JAMA.

[3] Lee M, Park S, Lee K (2020). Relationship between morbidity and health behavior in chronic diseases. J Clin Med.

[4] Loef M, Walach H (2012). The combined effects of healthy lifestyle behaviors on all cause mortality: a systematic review and meta-analysis. Prev Med.

[5] Desroches S, Lapointe A, Ratté S, Gravel K, Légaré F, Turcotte S (2013). Interventions to enhance adherence to dietary advice for preventing and managing chronic diseases in adults. Cochrane Database Syst Rev.

[6] Hartmann-Boyce J, Livingstone-Banks J, Ordóñez-Mena J (2021). Behavioural interventions for smoking cessation: an overview and network meta-analysis. Cochrane Database Syst Rev.

[7] Verma M, Hontecillas R, Tubau-Juni N, Abedi V, Bassaganya-Riera J (2018). Challenges in personalized nutrition and health. Front. Nutr.

[8] O'Sullivan A, Henrick B, Dixon B, Barile D, Zivkovic A, Smilowitz J, Lemay D, Martin W, German JB, Schaefer SE (2018). 21st century toolkit for optimizing population health through precision nutrition. Crit Rev Food Sci Nutr.

[9] Lippke S, Ziegelmann JP (2008). Theory-based health behavior change: developing, testing, and applying theories for evidence-based interventions. Appl Psychol.

[10] Davis R, Campbell R, Hildon Z, Hobbs L, Michie S (2015). Theories of behaviour and behaviour change across the social and behavioural sciences: a scoping review. Health Psychol Rev.

[11] Michie S, van Stralen MM, West R (2011). The behaviour change wheel: a new method for characterising and designing behaviour change interventions. Implement Sci.

[12] Vigneshvar S, Sudhakumari CC, Senthilkumaran B, Prakash H (2016). Recent advances in biosensor technology for potential applications—an overview. Front Bioeng Biotechnol.

[13] Peake JM, Kerr G, Sullivan JP (2018). A critical review of consumer wearables, mobile applications, and equipment for providing biofeedback, monitoring stress, and sleep in physically active populations. Front Physiol.

[14] Marcum JA (2020). Nutrigenetics/nutrigenomics, personalized nutrition, and precision healthcare. Curr Nutr Rep.

[15] Wynn RM, Adams KT, Kowalski RL, Shivega WG, Ratwani RM, Miller KE (2018). The patient in precision medicine: a systematic review examining evaluations of patient-facing materials. J Healthc Eng.

[16] The Theory and Techniques Tool.

[17] Biofeedback, psychology. NCBI Resources.

[18] Kondo K, Noonan KM, Freeman M, Ayers C, Morasco BJ, Kansagara D (2019). Efficacy of biofeedback for medical conditions: an evidence map. J Gen Intern Med.

[19] Celis-Morales C, Livingstone KM, Marsaux CFM, Forster H, O'Donovan CB, Woolhead C, Macready AL, Fallaize R, Navas-Carretero S, San-Cristobal R, Kolossa S, Hartwig K, Tsirigoti L, Lambrinou CP, Moschonis G, Godlewska M, Surwiłło A, Grimaldi K, Bouwman J, Daly EJ, Akujobi V, O'Riordan R, Hoonhout J, Claassen A, Hoeller U, Gundersen TE, Kaland SE, Matthews JNS, Manios Y, Traczyk I, Drevon CA, Gibney ER, Brennan L, Walsh MC, Lovegrove JA, Alfredo MJ, Saris WHM, Daniel H, Gibney M, Mathers JC (2015). Design and baseline characteristics of the Food4Me study: a web-based randomised controlled trial of personalised nutrition in seven European countries. Genes Nutr.

[20] Celis-Morales C, Livingstone KM, Marsaux CFM, Macready AL, Fallaize R, O'Donovan CB, Woolhead C, Forster H, Walsh MC, Navas-Carretero S, San-Cristobal R, Tsirigoti L, Lambrinou CP, Mavrogianni C, Moschonis G, Kolossa S, Hallmann J, Godlewska M, Surwiłło A, Traczyk I, Drevon CA, Bouwman J, van OB, Grimaldi K, Parnell LD, Matthews JNS, Manios Y, Daniel H, Martinez JA, Lovegrove JA, Gibney ER, Brennan L, Saris WHM, Gibney M, Mathers JC, Food4Me S (2016). Effect of personalized nutrition on health-related behaviour change: evidence from the Food4me European randomized controlled trial. Int J Epidemiol.

[21] Celis-Morales C, Livingstone KM, Petermann-Rocha F, Navas-Carretero S, San-Cristobal R, O'Donovan CB, Moschonis G, Manios Y, Traczyk I, Drevon CA, Daniel H, Marsaux CFM, Saris WHM, Fallaize R, Macready AL, Lovegrove JA, Gibney M, Gibney ER, Walsh M, Brennan L, Martinez JA, Mathers JC, Food4Me Study (2019). Frequent nutritional feedback, personalized advice, and behavioral changes: findings from the European Food4Me internet-based RCT. Am J Prev Med.

[22] McClure J (2002). Are biomarkers useful treatment aids for promoting health behavior change?: An empirical review. Am J Prev Med.

[23] Peters M, Godfrey C, McInerney P, Munn Z, Trico A, Khalil H, Aromataris E, Munn Z (2020). Chapter 11: Scoping reviews. JBI Manual for Evidence Synthesis.

[24] Gochman DS (1997). Handbook of Health Behavior Research I: Personal and Social Determinants.

[25] Conner M (2015). Health behaviors. International Encyclopedia of the Social & Behavioral Sciences (Second Edition).

[26] Page MJ, McKenzie JE, Bossuyt PM, Boutron I, Hoffmann TC, Mulrow CD, Shamseer L, Tetzlaff JM, Akl EA, Brennan SE, Chou R, Glanville J, Grimshaw JM, Hróbjartsson A, Lalu MM, Li T, Loder EW, Mayo-Wilson E, McDonald S, McGuinness LA, Stewart LA, Thomas J, Tricco AC, Welch VA, Whiting P, Moher D (2021). The PRISMA 2020 statement: an updated guideline for reporting systematic reviews. BMJ.

