# Supplementary material for: Using Biological Feedback to Promote Health Behavior Change in Adults: Protocol for a Scoping Review
Source: JMIR Res Protoc. 2022 Jan 18;11(1):e32579. doi: 10.2196/32579 (PMC8808341; doi:10.2196/32579)
Supplement: Multimedia Appendix 1 [file resprot_v11i1e32579_app1.docx]

Ovid MEDLINE(R) and Epub Ahead of Print, In-Process, In-Data-Review & Other Non-Indexed Citations, Daily and Versions(R) <1946 to June 01, 2021>

| # | Searches |
| --- | --- |
| 1 | biological phenomena/ |
| 2 | biomarkers/ |
| 3 | physiological phenomena/ |
| 4 | monitoring, physiologic/ |
| 5 | biological monitoring/ |
| 6 | body weight/ |
| 7 | risk assessment/ |
| 8 | basal metabolism/ |
| 9 | blood glucose/ |
| 10 | blood pressure/ |
| 11 | exp body composition/ |
| 12 | "body weights and measures"/ |
| 13 | exp "body fat distribution"/ |
| 14 | "body mass index"/ |
| 15 | "waist-hip ratio"/ |
| 16 | exp body size/ |
| 17 | exp blood cells/ |
| 18 | blood.fs. |
| 19 | urine.fs. |
| 20 | cholesterol/ |
| 21 | gastrointestinal microbiome/ |
| 22 | genetic predisposition to disease/ |
| 23 | genetic profile/ |
| 24 | genetic markers/ |
| 25 | genetic carrier screening/ |
| 26 | genetic testing/ |
| 27 | health status indicators/ |
| 28 | health status/ |
| 29 | heart rate/ |
| 30 | exp muscle strength/ |
| 31 | nutritional status/ |
| 32 | pulse/ |
| 33 | respiratory rate/ |
| 34 | vital signs/ |
| 35 | breath tests/ |
| 36 | glycated hemoglobin a/ |
| 37 | anthropometry/ |
| 38 | blood chemical analysis/ |
| 39 | health impact assessment/ |
| 40 | precision medicine/ |
| 41 | diagnostic tests, routine/ |
| 42 | exp respiratory function tests/ |
| 43 | exp spirometry/ |
| 44 | exp oximetry/ |
| 45 | blood pressure determination/ |
| 46 | "direct-to-consumer screening and testing"/ |
| 47 | exp disease susceptibility/ |
| 48 | exp exercise test/ |
| 49 | heart rate determination/ |
| 50 | hematologic tests/ |
| 51 | exp blood cell counts/ |
| 52 | physical examination/ |
| 53 | carbon monoxide/ |
| 54 | nutritional physiological phenomena/ |
| 55 | nutrition assessment/ |
| 56 | galvanic skin response/ |
| 57 | urinalysis/ |
| 58 | ethanol/ |
| 59 | "blood alcohol content"/ |
| 60 | exp "Diagnostic Imaging"/ |
| 61 | "diagnostic imaging".fs. |
| 62 | electrooculography/ |
| 63 | exp "Electrocardiography"/ |
| 64 | exp "Plethysmography"/ |
| 65 | cotinine/ |
| 66 | metabolic equivalent/ |
| 67 | ketones/ |
| 68 | ketone bodies/ |
| 69 | bone density/ |
| 70 | "absorptiometry, photon"/ |
| 71 | electric impedance/ |
| 72 | (biological adj3 (data or information or metric* or marker* or measure* or indicator* or risk*)).tw. |
| 73 | biomarker*.tw. |
| 74 | (blood adj3 analyte*).tw. |
| 75 | metabolite*.tw. |
| 76 | ("gut microbiome" or "gut microflora" or "gut bacteria" or "gut microbiota").tw. |
| 77 | nutrigenetic*.tw. |
| 78 | epigenetic*.tw. |
| 79 | "risk indicator*".tw. |
| 80 | "risk appraisal*".tw. |
| 81 | "health hazard appraisal*".tw. |
| 82 | (modifiable adj3 "risk factor*").tw. |
| 83 | ("health status" adj3 indicator*).tw. |
| 84 | glucose.tw. |
| 85 | (HgA1c or HbA1c or "h?emoglobin A1c").tw. |
| 86 | ("glycated h?emoglobin" or "glycosylated h?emoglobin").tw. |
| 87 | anthropom*.tw. |
| 88 | "blood pressure*".tw. |
| 89 | weight.tw. |
| 90 | BMI.tw. |
| 91 | "body mass index".tw. |
| 92 | "body measurement*".tw. |
| 93 | "body composition".tw. |
| 94 | "waist hip".tw. |
| 95 | "waist circumference".tw. |
| 96 | "carbon monoxide".tw. |
| 97 | ("genetic susceptibility" or "genetic predisposition").tw. |
| 98 | (genetic adj1 (risk* or test* or screen*)).tw. |
| 99 | (blood adj3 test*).tw. |
| 100 | ("lung function" adj3 test*).tw. |
| 101 | "exercise test".tw. |
| 102 | "muscle strength".tw. |
| 103 | "personali#ed risk".tw. |
| 104 | "heart rate".tw. |
| 105 | "lipid profile".tw. |
| 106 | cholesterol.tw. |
| 107 | "liver enzyme*".tw. |
| 108 | "galvanic skin response".tw. |
| 109 | "skin conductance response".tw. |
| 110 | (electrodermal adj1 (activity or response)).tw. |
| 111 | urinalysis.tw. |
| 112 | (ultrasound or ultrasonogr*).tw. |
| 113 | "computed tomogr*".tw. |
| 114 | electro#ardiogra*.tw. |
| 115 | (ekg or ecg).tw. |
| 116 | plethysmography.tw. |
| 117 | cotinine.tw. |
| 118 | spirometry.tw. |
| 119 | "breath test*".tw. |
| 120 | "risk assessment".tw. |
| 121 | ("precision nutrition" or "precision medicine").tw. |
| 122 | electrooculography.tw. |
| 123 | (ethanol or "blood alcohol").tw. |
| 124 | "pulmonary function test*".tw. |
| 125 | ketone*.tw. |
| 126 | ("beta-hydroxybutyrate" or "3-hydroxybutyric acid").tw. |
| 127 | ("bone density" or "dexa scan" or "dxa scan" or "dual energy x-ray absorptiometry").tw. |
| 128 | bod?pod.tw. |
| 129 | "underwater weigh*".tw. |
| 130 | "hydrostatic weigh*".tw. |
| 131 | hydrodensitometry.tw. |
| 132 | or/1-131 |
| 133 | feedback/ |
| 134 | feedback, psychological/ |
| 135 | communication/ |
| 136 | health communication/ |
| 137 | health education/ |
| 138 | patient education as topic/ |
| 139 | cues/ |
| 140 | genetic counseling/ |
| 141 | counseling/ |
| 142 | motivational interviewing/ |
| 143 | exp "correspondence as topic"/ |
| 144 | "behavior therapy"/ |
| 145 | therapy.fs. and lifestyle/ |
| 146 | wearable electronic devices/ |
| 147 | exp monitoring, ambulatory/ |
| 148 | exp self-testing/ |
| 149 | telephone/ |
| 150 | exp cell-phone/ |
| 151 | feedback*.tw. |
| 152 | ((communicat* or provide*) adj3 (results or information or risk*)).tw. |
| 153 | "cue* to action".tw. |
| 154 | ((tailored or personali#ed or individuali#ed) adj5 (communicat* or information)).tw. |
| 155 | (receiv* adj3 (results or information)).tw. |
| 156 | ((knowledge or knowing) adj3 result*).tw. |
| 157 | counsel*.tw. |
| 158 | coach*.tw. |
| 159 | advice.tw. |
| 160 | "motivational interviewing".tw. |
| 161 | (behavio?r* adj1 (therap* or intervention*)).tw. |
| 162 | ((tailored or personali#ed or individuali#ed) adj3 educat*).tw. |
| 163 | (education* adj3 (module or support or session)).tw. |
| 164 | (educate adj3 (individual* or patient*)).tw. |
| 165 | (received adj3 education*).tw. |
| 166 | ((patient or health) adj1 education).tw. |
| 167 | wearable*.tw. |
| 168 | biosensor*.tw. |
| 169 | "sensor technolog*".tw. |
| 170 | (device adj3 (monitor* or track*)).tw. |
| 171 | "smart device*".tw. |
| 172 | (self adj3 (monitor* or test*)).tw. |
| 173 | "ambulatory monitor*".tw. |
| 174 | ("personal* health" adj3 monitor*).tw. |
| 175 | messag*.tw. |
| 176 | (telephone* or cellphone* or cell-phone* or smartphone* or smart-phone* or "mobile phone*").tw. |
| 177 | ((month* or week*) adj3 support).tw. |
| 178 | ((interpreted or reported or informed) adj3 (measurement* or result* or data or level*)).tw. |
| 179 | or/133-178 |
| 180 | 132 and 179 |
| 181 | "blood glucose self-monitoring"/ |
| 182 | "blood pressure monitors"/ or "Blood Pressure Monitoring, Ambulatory"/ |
| 183 | ((home or self or continuous or ambulatory) adj3 ("blood pressure monitor*" or "glucose monitor*")).tw. |
| 184 | "flash glucose monitor*".tw. |
| 185 | ("hr monitor*" or "heart rate monitor*").tw. |
| 186 | "physiological feedback".tw. |
| 187 | "biological feedback".tw. |
| 188 | biofeedback.tw. |
| 189 | self-weigh*.tw. |
| 190 | "daily weigh*".tw. |
| 191 | (connected adj1 (glucometer* or scale*)).tw. |
| 192 | "smart scale*".tw. |
| 193 | (BIA adj3 scale*).tw. |
| 194 | (("bio?electric* impedance" adj3 (scale* or analysis)) or (bio?impedance adj3 (scale* or analysis))).tw. |
| 195 | or/181-194 |
| 196 | 180 or 195 |
| 197 | behavior/ |
| 198 | exp health behavior/ |
| 199 | behavior control/ |
| 200 | behavioral medicine/ |
| 201 | behavioral research/ |
| 202 | feeding behavior/ |
| 203 | health, knowledge, attitudes, practice/ |
| 204 | exp healthy lifestyle/ |
| 205 | exp health promotion/ |
| 206 | exp motivation/ |
| 207 | risk reduction behavior/ |
| 208 | self-efficacy/ |
| 209 | self-care/ |
| 210 | self-management/ |
| 211 | awareness/ |
| 212 | exp inhibition, psychological/ |
| 213 | "Treatment Adherence and Compliance"/ |
| 214 | Patient Compliance/ |
| 215 | patient participation/ |
| 216 | public health/ |
| 217 | public health practice/ |
| 218 | preventive medicine/ |
| 219 | prevention & control.fs. |
| 220 | preventive health services/ |
| 221 | exp primary prevention/ |
| 222 | secondary prevention/ |
| 223 | tertiary prevention/ |
| 224 | smoking prevention/ |
| 225 | harm reduction/ |
| 226 | treatment outcome/ and (lifestyle/ or psychology.fs.) |
| 227 | ((behavio?r* or lifestyle) adj3 (chang* or modif* or promot*)).tw. |
| 228 | "health behavio?r*".tw. |
| 229 | "healthy lifestyle".tw. |
| 230 | (self adj3 (care or management or efficacy)).tw. |
| 231 | awareness.tw. |
| 232 | ((risk or harm or "sedentary behavio?r") adj3 reduc*).tw. |
| 233 | "weight loss".tw. |
| 234 | "weight control".tw. |
| 235 | (smok* adj3 (behavio?r* or cessation or quit*)).tw. |
| 236 | "self regulat*".tw. |
| 237 | (motivated or motivation).tw. |
| 238 | (adherence or compliance).tw. |
| 239 | (prevention or preventive).tw. |
| 240 | "health promotion".tw. |
| 241 | (improv* adj3 (activit* or eating or diet* or health or fitness)).tw. |
| 242 | ((exercise or "physical activity" or diet* or eating or weight) adj3 (behavio?r* or chang* or maint* or motivat* or promot* or modif*)).tw. |
| 243 | "public health".tw. |
| 244 | or/197-243 |
| 245 | 196 and 244 |
| 246 | limit 245 to medline |
| 247 | 245 not 246 |
| 248 | randomized controlled trial.pt. |
| 249 | controlled clinical trial.pt. |
| 250 | randomi#ed.ab. |
| 251 | clinical trials as topic.sh. |
| 252 | randomly.ab. |
| 253 | trial.ti. |
| 254 | 248 or 249 or 250 or 251 or 252 or 253 |
| 255 | exp animals/ not humans.sh. |
| 256 | 254 not 255 |
| 257 | 246 and 256 |
| 258 | random*.tw. |
| 259 | trial.tw. |
| 260 | 258 or 259 |
| 261 | 247 and 260 |
| 262 | 257 or 261 |
