# Supplementary material for: Using Biological Feedback to Promote Health Behavior Change in Adults: Protocol for a Scoping Review
Source: JMIR Res Protoc. 2022 Jan 18;11(1):e32579. doi: 10.2196/32579 (PMC8808341; doi:10.2196/32579)
Supplement: Multimedia Appendix 2 [file resprot_v11i1e32579_app2.docx]

|  | Descriptions^a^ |
| --- | --- |
| Author(s) |  |
| Title |  |
| Year |  |
| Biological measure |  Blood pressure   Carbon monoxide   Genetics   Glucose   Weight  ... |
| Targeted behavior |  Alcohol use reduction/cessation   Diet   Physical activity   Smoking reduction/cessation   Sun protection  ... |
| Targeted health-related outcome/Intent of intervention |  Drug use cessation   Glycemic control   Lipid (cholesterol, triglycerides) management   Mental health improvement   Weight management  ... |
| Domain |  Cancer   Cardiovascular disease   Diabetes   Osteoporosis   Substance abuse  ... |
| Biological measure measurement |  Self-measurement   Clinic/other |
| Feedback platform |  In-person   Monitoring device   Mobile app   Phone call  ... |
| Format of feedback |  Graph   Image   Number   Summarized (e.g., risk assessment, report)  ... |
| Behavior change theory |  Health Belief Model   Social Cognitive Theory   Theory of Planned Behavior   Transtheoretical Model  ... |
| Is biological feedback being used as the solo intervention, or is it part of a multi-component intervention? |  Solo intervention   Multi-component intervention |
| Is the study design meant to test the effect of biological feedback as an intervention strategy? |  Yes   No |

^a^ Includes examples of categories which will develop as concepts are revealed through results
